# Safety and tolerability of short‐term infusions of intravenous lacosamide in pediatric patients with epilepsy: An open‐label, phase 2/3 trial

**DOI:** 10.1002/epi4.12682

**Published:** 2023-01-18

**Authors:** Mark Kristof Farkas, Cynthia Beller, Ali Bozorg, Carrie McClung, Robert Roebling, Tanisia Yates, Nancy Yuen, Iryna Makedonska

**Affiliations:** ^1^ First Department of Pediatrics Semmelweis University Budapest Hungary; ^2^ UCB Pharma Morrisville North Carolina USA; ^3^ UCB Pharma Monheim am Rhein Germany; ^4^ Dnipro City Pediatric Clinical Hospital Dnipro Ukraine

**Keywords:** antiseizure medication, focal seizure, intravenous lacosamide, pediatric, primary generalized tonic–clonic seizures

## Abstract

**Objective:**

The objective of this study is to evaluate the safety and tolerability of intravenous (IV) lacosamide infusion in patients aged ≥1 month to <17 years with epilepsy.

**Methods:**

This Phase 2/3 open‐label trial (EP0060; NCT02710890) enrolled patients in two age cohorts (cohort 1: ≥8 to <17 years; cohort 2: ≥1 month to <8 years). Eligible patients were receiving oral lacosamide as adjunctive treatment or monotherapy (in an open‐label long‐term trial or by prescription) or were not receiving lacosamide before enrolment. Patients initiated IV lacosamide (2‐12 mg/kg/day or 100‐600 mg/day; 15‐60 minutes infusion) as a replacement for oral lacosamide or as adjunctive treatment. The primary outcomes were treatment‐emergent adverse events (TEAEs) and discontinuations due to TEAEs.

**Results:**

In total, 103 patients were enrolled and completed the trial; 55 patients were included in cohort 1 (≥8 to <17 years), 48 in cohort 2 (≥1 month to <8 years). During the 4 weeks before screening, 74 (71.8%) patients had focal seizures, 12 (11.7%) had generalized seizures, and two (1.9%) had unclassified seizures. Most patients (74 [71.8%]) initiated lacosamide as adjunctive IV treatment. The mean overall duration of exposure to IV lacosamide was 1.18 days. Seventy‐nine (76.7%) patients had one IV lacosamide infusion, 20 (19.4%) had two, one (1.0%) had three, and three (2.9%) had 10 infusions. Overall, five (4.9%) patients had a total of seven TEAEs. The only TEAEs reported in two or more patients were increased blood triglycerides (two [1.9%]). No serious or severe TEAEs were reported, and no patients discontinued due to TEAEs. No TEAEs were considered drug‐related by the investigator. No consistent or clinically relevant treatment‐related changes from baseline were observed for hematology, clinical chemistry parameters, vital signs, or 12‐lead electrocardiograms.

**Significance:**

IV lacosamide was generally well tolerated in pediatric patients (≥1 month to <17 years) with epilepsy, and no new safety concerns were identified.

**Key Points**

- Safety and tolerability of IV lacosamide infusions were evaluated in 103 pediatric patients (≥1 month to <17 years of age) with epilepsy.
- Seventy‐nine (76.7%) patients had one IV lacosamide infusion, 20 (19.4%) had two, one (1.0%) had three, and three (2.9%) had 10 infusions.
- The only TEAEs reported in ≥2 patients were increased blood triglycerides. No serious, severe, or drug‐related TEAEs were reported.
- No consistent or clinically relevant changes were observed for hematology, clinical chemistry parameters, vital signs, or 12‐lead ECGs.
- IV lacosamide was generally well tolerated, and no new safety concerns were identified.

## 1 INTRODUCTION

Lacosamide is an antiseizure medication (ASM). It is a functionalised amino acid that selectively enhances slow inactivation of neuronal voltage‐gated sodium channels.^1^ Lacosamide is indicated for the treatment of focal (partial‐onset) seizures in patients ≥1 month of age in the United States, and in patients ≥2 years of age in the European Union. Lacosamide is also indicated as adjunctive therapy for primary generalized tonic–clonic seizures in patients ≥4 years of age in the United States and the European Union.^2^, ^3^ The intravenous (IV) formulation of lacosamide may be used when oral administration (tablet or oral solution) is temporarily not feasible. The recommended infusion duration is 30‐60 minutes.^2^, ^3^

Several trials have investigated the efficacy and safety of oral formulations of lacosamide in pediatric patients. The efficacy and tolerability of adjunctive lacosamide in patients aged 4 to 17 years with uncontrolled focal seizures was demonstrated in a Phase 3 double‐blind trial.^4^ Another Phase 3 double‐blind trial evaluated adjunctive lacosamide in patients ≥1 month to <4 years with uncontrolled focal seizures; although the primary efficacy endpoint was not met, lacosamide was generally well tolerated with an acceptable safety profile.^5^ Data from an open‐label, fixed‐titration trial support the safety and tolerability of adjunctive lacosamide in patients aged 6 months to 17 years with focal seizures.^6^ Eligible patients completing the double‐blind trials or the fixed‐titration trial could enroll in the respective open‐label extension (EP0034; NCT01964560 or SP848; NCT00938912).

The safety and tolerability of IV lacosamide has been established in adults with focal seizures.^7^, ^8^, ^9^ IV lacosamide has been assessed in pediatric patients with epilepsy in small retrospective and open‐label studies only. In a retrospective study of 47 critically ill children ≤12 years of age with focal or generalized seizures, IV lacosamide was well tolerated with mild and reversible adverse events.^10^ Among 18 children hospitalized because of increased seizure frequency, 16 had a >50% reduction in seizure frequency for 48 hours after initiation of IV lacosamide and daily oral maintenance lacosamide.^10^ Other studies in pediatric patients with epilepsy and critically ill children have indicated that IV lacosamide is well tolerated.^11^, ^12^

At the time that this trial (EP0060; NCT02710890) was conducted, the IV formulation of lacosamide was indicated in the United States for temporary use in patients aged 17 years and older only. The primary objective of this trial was to evaluate the safety and tolerability of IV lacosamide infusions in pediatric patients with epilepsy ≥1 month to <17 years of age.

## 2 METHODS

EP0060 was a Phase 2/3, multicentre, open‐label trial. The trial was conducted in accordance with the International Council for Harmonization Good Clinical Practice requirements, the ethical tenets that have their origin in the principles of the Declaration of Helsinki, and the local laws of the countries involved. Written informed consent was provided by the patient or their legal representative(s) before enrolment. Legal guardians were provided with a written explanation of the study design prior to providing consent for their child's participation. The study protocol, amendments, and patient informed consent were reviewed and approved by a national, regional, or Independent Ethics Committee or Institutional Review Board.

Approximately 100 patients were planned for enrolment in two age‐based cohorts: cohort 1 was planned to include at least 40 patients aged ≥8 to <17 years, and cohort 2 was planned to include approximately 44 patients aged from ≥1 month to <8 years. Enrolment began with cohort 1. When the trial was initially conceived, the planned age range for cohort 2 was ≥4 to <8 years. A protocol amendment dated April 30, 2018 lowered the minimum age to 1 month based on newly available safety and efficacy data indicating no specific risk in this age group. After completion of the first 20 patients in cohort 1, an Independent Data Monitoring Committee (IDMC) reviewed the safety and tolerability data and recommended that additional patients could be enrolled in cohort 1 and enrolment into cohort 2 could be initiated.

### 2.1 Patient eligibility

Patients could be enrolled if they were ≥1 month to <17 years of age, had a diagnosis of epilepsy with focal seizures or primary generalized tonic–clonic seizures, weighed ≥4 kg, and were considered an acceptable candidate for venipuncture and IV infusion. In addition, eligible patients were (i) receiving oral lacosamide as adjunctive treatment or monotherapy in an open‐label long‐term trial (SP848 or EP0034); (ii) receiving prescribed oral lacosamide from a commercial supply as adjunctive treatment or monotherapy; or (iii) not receiving lacosamide treatment before enrolment (would receive IV lacosamide as a new adjunctive treatment in EP0060).

For patients who were receiving lacosamide upon enrolment, oral lacosamide must have been administered at a dose of 2‐12 mg/kg/day (in patients <50 kg) or 100‐600 mg/day (in patients ≥50 kg) for ≥2 weeks before screening. Oral lacosamide dose must have been stable for at least 3 days before the first lacosamide infusion. Patients initiating lacosamide had to be on a stable dosage regimen of at least one ASM, which must have been kept constant for ≥2 weeks before screening, and must not have received lacosamide within the 3 months before screening. Further details of eligibility and withdrawal criteria may be found in Supporting Information (Appendix S1).

### 2.2 Lacosamide dosing

The trial consisted of a screening and/or baseline period of up to 7 days; a treatment period; a final visit (1 day); and a safety follow‐up via telephone over a period of 1‐3 days. Patients received IV lacosamide based on clinical need or elective administration. Clinical need administration applied to patients who needed to undergo a procedure and were being treated at an epilepsy monitoring unit or healthcare facility or were in other situations where IV administration was clinically appropriate and oral administration was not feasible (e.g., surgery). For these patients, the maximum number of IV lacosamide doses was 10 (administered twice daily with an interval of approximately 12 hours, over a duration of ≤5 days). Elective administration applied to patients who were taking oral lacosamide (or any enteric lacosamide administration, e.g., by feeding tube), and elected to receive IV lacosamide at an epilepsy monitoring unit or healthcare facility. For these patients, a maximum of two IV lacosamide doses were permitted (over approximately 24 hours). Patients who only required one IV lacosamide infusion could complete all the trial periods in 1 day, provided there was sufficient time for all examinations and the final visit assessments.

Patients receiving lacosamide before this trial received IV lacosamide (as adjunctive treatment or monotherapy) as a replacement for oral lacosamide in a twice‐daily regimen at the same stable daily dose they had been receiving before the present trial: 2‐12 mg/kg/day or 100‐600 mg/day, with a maximum dose of 12 mg/kg/day or 600 mg/day, whichever was lower. Patients initiating lacosamide received IV lacosamide as adjunctive treatment only (initiation of IV lacosamide monotherapy was not permitted) in a twice‐daily regimen at a dose of 2 mg/kg/day for patients weighing <50 kg, and 100 mg/day for patients weighing ≥50 kg (the dose was to remain unchanged for the duration of the treatment period).

The first 20 patients enrolled in cohort 1 had a target IV lacosamide infusion duration of 30‐60 minutes. Following IDMC recommendations, the remaining patients in cohort 1 had a target infusion duration of 15‐30 minutes, provided that the investigator considered that they would directly benefit from an increased infusion rate. Patients who would not directly benefit from a faster infusion (in the opinion of the investigator) had a target infusion duration of 30‐60 minutes. For the first 20 patients in cohort 2, IV lacosamide was infused over a target duration of 30‐60 minutes whenever possible. Once these patients had completed the trial, a second IDMC review took place and recommended that a 30‐60 minutes infusion duration should continue to be used in cohort 2, as no previous participants had a clinical need necessitating a dose infusion time of 15‐30 minutes.

Patients who entered EP0060 from an open‐label, long‐term trial (SP848 or EP0034) suspended their participation in that trial temporarily to receive IV lacosamide. Upon completion of the EP0060 trial, eligible patients who had received prescribed lacosamide from commercial supply or who were not receiving lacosamide before enrolment had the option to continue oral lacosamide in another open‐label trial (SP848).

### 2.3 Outcomes

The primary outcomes were treatment‐emergent adverse events (TEAEs), reported spontaneously by the patient and/or caregiver or observed by the investigator, and discontinuations due to TEAEs. Other safety outcomes included changes in 12‐lead electrocardiograms (ECGs), vital sign measurements (blood pressure and pulse rate), physical examinations, and neurological examinations. The Safety Set (SS) was defined as all patients who received at least one dose of lacosamide (oral and/or IV) in this open‐label trial. The SS‐IV was defined as all patients in the SS who received at least one dose of IV lacosamide and was the primary analysis set for the safety data.

## 3 RESULTS

### 3.1 Patient disposition and baseline characteristics

This open‐label trial was conducted between May 2017 and June 2019; 103 patients were enrolled and completed the trial (SS; 77 from Europe, 26 from North America). Most patients (96 [93.2%]) were White. Fifty‐five patients were ≥8 to <17 years of age (cohort 1) and 48 were ≥1 month to <8 years of age (cohort 2). Patients had a mean age of 8.6 years, and 57 (55.3%) were female (Table 1). During the 4 weeks before the screening visit, 74 (71.8%) patients had focal seizures, 12 (11.7%) had generalized seizures, and two (1.9%) had unclassified seizures (patients could have had more than one type of seizure, and some were seizure‐free during this time period). The most common concomitant ASMs taken during the trial (≥20% of all patients) were levetiracetam (43 [41.7%]) and valproic acid (35 [34.0%]).

**TABLE 1:** Baseline demographics, medical conditions, epilepsy characteristics, concomitant ASMs, and target infusion duration (SS‐IV)

Characteristic	Patients ≥1 mo to <8 y of age (N = 48)	Patients ≥8 to < 17 y of age (N = 55)	All patients (N = 103)
Age, mean (SD), years	3.84 (2.33)	12.66 (2.41)	8.55 (5.01)
Female, n (%)	26 (54.2)	31 (56.4)	57 (55.3)
Patients entering EP0060, n (%)			
From an open‐label long‐term trial^a^	0	3 (5.5)	3 (2.9)
Receiving prescribed oral lacosamide	6 (12.5)	20 (36.4)	26 (25.2)
Not receiving lacosamide before enrolment	42 (87.5)	32 (58.2)	74 (71.8)
Previous and ongoing medical conditions in ≥10% of all patients, n (%)			
Any previous and ongoing medical conditions	41 (85.4)	42 (76.4)	83 (80.6)
Mental retardation	21 (43.8)	9 (16.4)	30 (29.1)
Cerebral palsy	13 (27.1)	8 (14.5)	21 (20.4)
Hypokinesia	15 (31.3)	1 (1.8)	16 (15.5)
Speech disorder developmental	11 (22.9)	2 (3.6)	13 (12.6)
History of epilepsy			
Time since first epileptic seizure, mean (SD), years	2.52 (2.02)	6.61 (4.64)^b^	4.68 (4.17)^c^
Age at diagnosis, mean (SD), years	1.69 (1.71)	6.29 (4.51)	4.15 (4.17)
Seizure classification history during 4 weeks before screening,^d^ n (%)			
Any partial‐onset seizures (*focal seizures*)	44 (91.7)	30 (54.5)	74 (71.8)
Simple partial (*focal aware*)	2 (4.2)	6 (10.9)	8 (7.8)
Complex partial (*focal impaired awareness*)	21 (43.8)	18 (32.7)	39 (37.9)
Partial evolving to secondary generalized (*focal to bilateral tonic–clonic*)	26 (54.2)	13 (23.6)	39 (37.9)
Any generalized seizures	1 (2.1)	11 (20.0)	12 (11.7)
Absence	0	1 (1.8)	1 (1.0)
Myoclonic	0	1 (1.8)	1 (1.0)
Clonic	1 (2.1)	0	1 (1.0)
Tonic	1 (2.1)	2 (3.6)	3 (2.9)
Tonic–clonic	0	7 (12.7)	7 (6.8)
Atonic	0	1 (1.8)	1 (1.0)
Unclassified epileptic seizures	2 (4.2)	0	2 (1.9)
Any concomitant ASMs, n (%)^e^	48 (100)	54 (98.2)	102 (99.0)
Concomitant ASMs, taken by ≥10% of all patients, n (%)			
Levetiracetam	18 (37.5)	25 (45.5)	43 (41.7)
Valproic acid	24 (50.0)	11 (20.0)	35 (34.0)
Carbamazepine	11 (22.9)	8 (14.5)	19 (18.4)
Oxcarbazepine	4 (8.3)	9 (16.4)	13 (12.6)
Topiramate	5 (10.4)	7 (12.7)	12 (11.7)
Lacosamide^f^	1 (2.1)	10 (18.2)	11 (10.7)
Target infusion duration, n (%)			
15–30 min	8 (16.7)	14 (25.5)	22 (21.4)
30–60 min	40 (83.3)	41 (74.5)	81 (78.6)

Abbreviations: ASM, antiseizure medication; IV, intravenous; SD, standard deviation; SS‐IV, intravenous Safety Set.

^a^ Trial SP848 or EP0034.

^b^ n = 54.

^c^ n = 102.

^d^ Patients could have more than one response in a classification level and/or category; seizure types are listed per the International League Against Epilepsy 1981 classification, with the newer terminology provided in parentheses.^3^, ^4^

^e^ Concomitant medications are medications taken on ≥1 day in common with IV lacosamide during the treatment period.

^f^ Lacosamide was reported as a concomitant ASM in patients who replaced one of their two daily oral doses with IV lacosamide.

### 3.2 Lacosamide exposure

**FIGURE 1:**
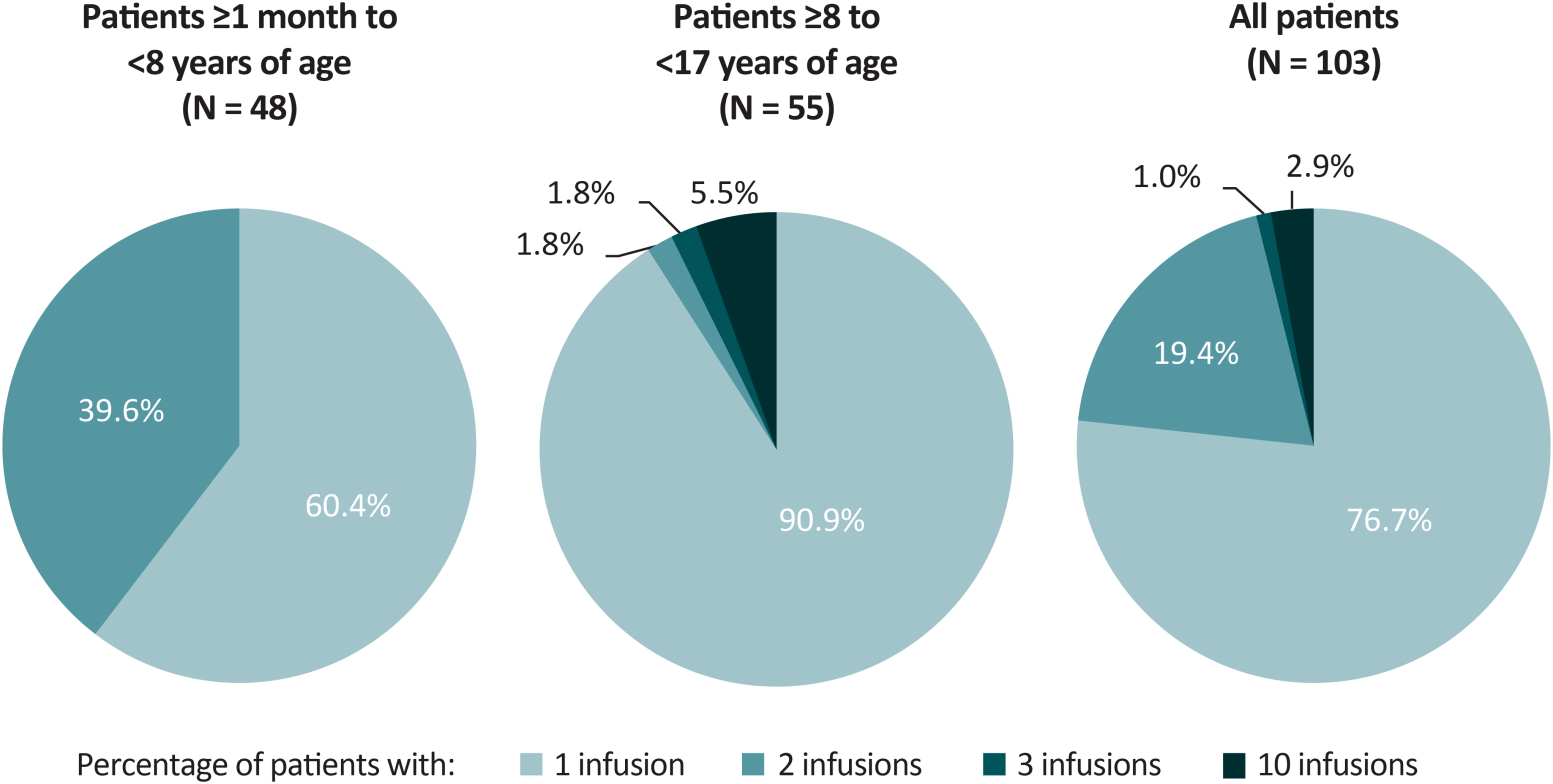
Number of infusions by cohort and overall (SS‐IV). SS‐IV, intravenous safety set

Most patients (74 [71.8%]) initiated lacosamide as adjunctive IV treatment upon enrolment, 26 (25.2%) received IV lacosamide as a replacement for prescribed oral lacosamide from a commercial supply, and three (2.9%) patients received IV lacosamide as a replacement for oral lacosamide received in another open‐label, long‐term trial. The mean overall duration of exposure to IV lacosamide was 1.18 days (median: 1 day; range: 1‐5 days; standard deviation [SD]: 0.71). The mean duration of exposure was 1.10 days (range: 1.0‐2.0 days; SD: 0.31) for patients ≥1 month to <8 years of age, and 1.25 days (range: 1.0–5.0 days; SD: 0.93) for patients ≥8 years to <17 years of age. Most (81 [78.6%]) patients had a target IV lacosamide infusion duration of 30‐60 minutes rather than 15‐30 minutes (22 [21.4%]). Seventy‐nine (76.7%) patients had one IV lacosamide infusion, 20 (19.4%) had two infusions, one (1.0%) had three infusions, and three (2.9%) had 10 infusions (Figure 1). No patients in the ≥1 month to <8 years age cohort received more than two infusions.

### 3.3 Safety and tolerability of IV lacosamide

A total of seven TEAEs were reported in five (4.9%) patients following treatment with IV lacosamide (Table 2). No serious TEAEs, severe TEAEs, or discontinuation due to TEAEs were reported. No TEAEs were considered drug‐related by the investigator, and no deaths were reported during the trial.

**TABLE 2:** Treatment‐emergent adverse events (SS‐IV)

	Patients ≥1 mo to <8 y of age (N = 48)	Patients ≥8 to < 17 y of age (N = 55)	All patients (N = 103)
Any TEAEs, n (%)	3 (6.3)	2 (3.6)	5 (4.9)
TEAEs reported during the post‐IV treatment period,^a^ n (%)			
Blood triglycerides increased	0	2 (3.6)	2 (1.9)
Blood cholesterol increased	0	1 (1.8)	1 (1.0)
Functional gastrointestinal disorder	1 (2.1)	0	1 (1.0)
Pyrexia	1 (2.1)	0	1 (1.0)
Respiratory tract infection	1 (2.1)	0	1 (1.0)
Respiratory tract infection viral	1 (2.1)	0	1 (1.0)

Abbreviations: IV, intravenous; SS‐IV, intravenous Safety Set; TEAE, treatment‐emergent adverse event.

^a^ Medical Dictionary for Regulatory Activities Version 16.1 Preferred Term.

The only TEAEs reported in two or more patients were increased blood triglycerides. One event occurred in a 10‐year‐old male who had a blood triglyceride level of 1.72 mmol/L at screening and 2.52 mmol/L at the final visit (normal range: 0.27–1.55 mmol/L). This TEAE was considered moderate in severity. The patient was taking oral lacosamide and levetiracetam at baseline and had discontinued oxcarbazepine 8 days previously. The other event occurred in a 12‐year‐old female who had a blood triglyceride level of 1.45 mmol/L at screening, 0.90 mmol/L at baseline, and 1.71 mmol/L at the final visit (normal range: 0.42–1.47 mmol/L). This TEAE was considered mild in severity. The patient was taking oral lacosamide and levetiracetam at baseline. Of note, triglyceride levels rise after eating. Patients were not required to fast prior to assessment of lipid panel.

Mean values for the majority of hematology and clinical chemistry parameters remained within the normal ranges for the duration of the trial (Appendix S1). No consistent or clinically relevant changes from baseline were observed in vital sign parameters (Appendix S1, Table S1). There were no treatment‐emergent clinically significant ECG findings, and no ECG‐related TEAEs were reported. None of the relatively small changes from baseline in 12‐lead ECG parameters (heart rate, QT interval, QT interval corrected for heart rate [QTcB and QTcF]) appeared to be clinically relevant. Mean changes from baseline to visit 2 and the final visit were small and similar between cohorts for PR interval, QRS duration, and QTcB and QTcF (Table S2). At all post‐baseline time points, there was no evidence of QT, QTcB, or QTcF prolongation following treatment with lacosamide (Table S2).

## 4 DISCUSSION

This open‐label, multicentre trial is the largest prospective investigation of the safety and tolerability of IV lacosamide in pediatric patients with epilepsy. IV lacosamide (2–12 mg/kg/day or 100‐600 mg/day) was generally well tolerated in patients ≥1 month to <17 years of age, and no new safety concerns were identified. Nearly 72% of patients had not received lacosamide treatment in the 3 months preceding the trial, and most of the remaining patients were receiving oral lacosamide from a commercial supply. There was a mean overall exposure of IV lacosamide of 1.18 days, and most patients received one dose as a 30‐ to 60‐minute infusion.

Bioequivalence of IV and oral lacosamide has been established in adults.^13^ In oral and IV lacosamide pediatric pharmacokinetic modeling, simulations of IV lacosamide infused over 15‐30 minutes resulted in similar exposure to oral administration.^14^ Using weight‐based dosing adaptations, it was predicted that the lacosamide concentration at steady state in children would be similar to that in adults for both oral and IV lacosamide. Based on PK modeling, the use of IV lacosamide was expected to be safe in pediatric patients down to 4 years of age.^14^ Therefore, enrolment of patients aged ≥4 to <17 years in two age‐based cohorts was initially planned, with initiation of the younger cohort subject to an IDMC review. During the course of the trial, new safety information for lacosamide in patients aged ≥1 month to 4 years was published.^10^, ^12^ As no specific risks were identified for this age group, the minimum age limit for the trial was lowered to ≥1 month.

As has been previously observed in studies of IV lacosamide in pediatric patients with epilepsy and in critically ill children, TEAEs were reported in few patients.^2^, ^10^, ^15^ The observed safety profile is consistent with the known safety profile of IV lacosamide in adults^7^, ^8^, ^9^ and of oral lacosamide in pediatric patients.^4^, ^5^, ^6^ No severe or serious TEAEs or discontinuation due to TEAEs were reported, and no TEAEs were considered related to lacosamide by the investigator. Two patients had TEAEs of increased blood triglycerides. Given the short duration of the trial, assessment of the lipid panel in a non‐fasting state, concomitant treatment with other ASMs, and elevated baseline triglycerides in one of the two patients, these changes in lipid levels are likely not related to treatment with IV lacosamide and are not clinically significant. Further, a post hoc analysis of serological data from a randomized monotherapy trial showed no change from baseline in serum lipid levels following 12 months of oral lacosamide therapy.^16^ ECG outcomes showed no clinically relevant changes. This is consistent with observations from a retrospective trial of critically ill children with focal or generalized seizures treated with IV lacosamide, in which no cardiac events were noted in 37 children who had continuous ECG monitoring before, during, and after infusion.^10^

The design of this trial was intended to maximize the available pool of patients by allowing for entry of individuals who were receiving oral lacosamide as adjunctive treatment or monotherapy (in an open‐label long‐term trial or by prescription), in addition to those who initiated IV lacosamide as adjunctive treatment following enrolment. Patients with ongoing oral lacosamide treatment were on a stable dose.

### 4.1 Limitations

This trial is limited by its open‐label, uncontrolled design with no comparator or placebo group. The patient population was mostly White; the results may not be generalizable across racial groups. However, no relevant differences in the pharmacokinetics of lacosamide have been observed between healthy Asian, Black, and Caucasian individuals.^2^ Given the study design, some drug‐related TEAEs may not have been detected. The study did not explore prolonged periods of infusion with IV lacosamide; longer‐term administration of IV treatment may be required for critically ill children or those who cannot tolerate oral medications for several days or longer.

Overall, IV lacosamide had an acceptable tolerability profile in pediatric patients ≥1 month to <17 years of age with epilepsy and focal seizures or primary generalized tonic–clonic seizures. No new safety concerns were identified.

## AUTHOR CONTRIBUTIONS

Mark Kristof Farkas and Iryna Makedonska were involved in execution of the trial as study investigators. Cynthia Beller was involved in analysis and interpretation of the data. Ali Bozorg and Nancy Yuen were involved in study design, and analysis and interpretation of the data. Carrie McClung and Robert Roebling were involved in study execution, and analysis and interpretation of the data. Tanisia Yates was involved in study design and execution. All authors critically reviewed the manuscript and approved the final version for submission.

## CONFLICTS OF INTEREST

Mark Kristof Farkas and Iryna Makedonska each report no conflicts of interest. Cynthia Beller, Ali Bozorg, Carrie McClung, Robert Roebling, Tanisia Yates, and Nancy Yuen are employees of UCB Pharma.

## ETHICAL APPROVAL

We confirm that we have read the Journal's position on issues involved in ethical publication and affirm that this report is consistent with those guidelines.

## Supporting information


Appendix S1–S2

## Data Availability

Underlying data from this manuscript may be requested by qualified researchers 6 months after product approval in the United States and/or Europe, or global development is discontinued, and 18 months after trial completion. Investigators may request access to anonymised individual patient‐level data and redacted trial documents which may include analysis‐ready datasets, study protocol, annotated case report form, statistical analysis plan, dataset specifications, and clinical study report. Prior to use of the data, proposals need to be approved by an independent review panel at www.Vivli.org and a signed data sharing agreement will need to be executed. All documents are available in English only, for a prespecified time, typically 12 months, on a password protected portal.
