# Supplementary material for: Safety and tolerability of short‐term infusions of intravenous lacosamide in pediatric patients with epilepsy: An open‐label, phase 2/3 trial
Source: Epilepsia Open. 2023 Jan 18;8(1):146–53. doi: 10.1002/epi4.12682 (PMC9977742; doi:10.1002/epi4.12682)
Supplement: Supplementary file 1 — Appendix S1–S2 [file EPI4-8-146-s001.docx]

**SUPPORTING INFORMATION**

# Appendix S1

## Methods

## Additional eligibility criteria

Exclusion criteria included previous treatment with IV lacosamide in EP0060; any medical, neurological, or psychiatric condition that, in the opinion of the investigator, could have jeopardised the patient’s health or compromised their ability to participate in EP0060; clinically significant hypotension or bradycardia; and treatment with monoamine oxidase A inhibitors. Patients ≥6 years of age were excluded if they had a lifetime history of suicide attempt, or suicidal ideation in the past 6 months as indicated by positive responses to either Question 4 or Question 5 of the Columbia Suicide Severity Rating Scale at screening.

Patients receiving oral lacosamide in an open-label long-term trial were not permitted to enrol in EP0060 if they had any ongoing adverse event that could have compromised their ability to participate, or if they had met any of the criteria for required withdrawal from the long-term trial. Patients receiving prescribed oral lacosamide from commercial supply and those not currently receiving lacosamide were excluded from EP0060 if they had a medical condition that could reasonably be expected to interfere with drug absorption, distribution, metabolism, or excretion; a known hypersensitivity to any component of the trial medication; or were a female of childbearing potential and did not practise an acceptable method of contraception for the duration of participation in EP0060.

Additional exclusion criteria included creatinine clearance less than 30 mL/min; a clinically relevant ECG abnormality (ie, second- or third-degree heart block at rest or a QT prolongation greater than 450 ms); haemodynamically significant heart disease (eg, heart failure) or an arrhythmic heart condition requiring medical therapy; a known history of severe anaphylactic reaction or serious blood dyscrasias; an acute or sub-acutely progressive central nervous system disease; or a known cardiac sodium channelopathy, such as Brugada syndrome.

Patients were not permitted to enrol if lacosamide was intended for treatment of generalised convulsive status epilepticus; if they had epilepsy secondary to a progressing cerebral disease or any other progressive or neurodegenerative disease (malignant brain tumour or Rasmussen syndrome); exclusively typical absence (Type IIA1) or atypical absence (Type IIA2) seizures; or a diagnosis of Dravet’s syndrome. In addition, patients were excluded if they had >2 × upper limit of normal (ULN) of any of the following: alanine aminotransferase, aspartate aminotransferase, alkaline phosphatase, or >ULN total bilirubin (≥1.5 × ULN total bilirubin if known Gilbert’s syndrome). If the patient had elevations only in total bilirubin that were >ULN and <1.5 × ULN, bilirubin should be fractionated to identify possible undiagnosed Gilbert’s syndrome (ie, direct bilirubin <35%).

## Trial withdrawal criteria

Patients were free to withdraw from EP0060 at any time, without prejudice to their continued care. Patients were withdrawn if they had intolerable adverse events and/or adverse events associated with IV administration that precluded further participation; if they required more than 10 IV lacosamide doses; if withdrawal was requested by the sponsor or a regulatory agency; if they had a corrected QT interval (QTc) ≥500 ms (confirmed by a cardiologist); if they became pregnant; if they developed a second- or third-degree atrioventricular block or another clinically relevant change in medical condition (or ECG); if they were unwilling/unable to continue; if the parent/legal guardian was unwilling/unable to allow continued trial participation; or if the investigator decided that trial withdrawal was in the patient’s best interests. Patients ≥6 years of age were withdrawn if they had actual suicidal ideation since last visit as indicated by a positive response (“Yes”) to either Question 4 or Question 5 of the “Children’s Since Last Visit” version of the Columbia Suicide Severity Rating Scale.

Patients may have been withdrawn if they experienced generalised convulsive status epilepticus; had any clinically relevant change in medical or psychiatric condition; required a medication that was not permitted by the protocol; or were non-compliant with EP0060 procedures or medication. Patients may also have been withdrawn if they initiated adjunctive lacosamide treatment in EP0060 and required a change in lacosamide dose during the treatment period, or if they were electively administering lacosamide and required more than two IV doses (if there was a clinical need to administer more than two IV doses as determined by the investigator, the patient may have remained in the trial).

## Results

## Haematology

Low percentages of patients overall reported worsening shifts from baseline to the end of the IV treatment period across haematology parameters.

- Shifts from normal at baseline to low at the end of IV treatment were observed for ≤5.8% of patients for platelets, haematocrit, haemoglobin, erythrocytes, eosinophils, lymphocytes, lymphocytes/leukocytes, neutrophils, neutrophils/leukocytes, and leukocytes.
- Shifts from normal at baseline to high at the end of IV treatment were observed for ≤10.7% of patients for haematocrit, haemoglobin, erythrocytes, eosinophils, eosinophils/leukocytes, lymphocytes/leukocytes, monocytes, monocytes/leukocytes, neutrophils, neutrophils/leukocytes, and leukocytes.
- Shifts from low at baseline to high at the end of IV treatment were observed for ≤1.0% of patients for lymphocytes/leukocytes and neutrophils/leukocytes.
- A shift from high at baseline to low at the end of IV treatment were observed for ≤1.0% of patients for haematocrit.

## Clinical chemistry

Low percentages of patients overall reported worsening shifts from baseline to the end of the IV treatment period across clinical chemistry parameters.

- Shifts from normal at baseline to low at the end of IV treatment were observed for ≤10.7% of patients for bicarbonate, calcium, creatinine, cholesterol, bilirubin, glucose, urate, and protein.
- Shifts from normal at baseline to high at the end of IV treatment were observed for ≤13.6% of patients for chloride, potassium, phosphate, sodium, alanine transaminase, aspartate aminotransferase, gamma-glutamyl transferase, creatinine, urea nitrogen, cholesterol, triglycerides, glucose, urate, and albumin.
- No shifts from low at baseline to high at the end of IV treatment were observed.
- No shifts from high at baseline to low at the end of IV treatment were observed.

## Vital signs

Mean changes from baseline through visit 2 and at the final visit for systolic blood pressure, diastolic blood pressure, and pulse rate values were small and similar between cohorts (Table S1). There were no patients with visit 3 values for cohort 2 (≥1 month to <8 years of age), and the mean changes from baseline at visit 3 were highly variable for cohort 1 (≥8 to <17 years of age) given the small number of patients (n = 5) at the visit. No vital signs–related TEAEs were reported.

**Table S1** Mean change from baseline in vital signs (SS-IV)

| **Change from baseline, mean (SD)** | | **Patients ≥1 month to  <8 years of age (N = 48)^a,b^** | **Patients ≥8 to  <17 years of age  (N = 55)^c,d^** |
| --- | --- | --- | --- |
| Systolic blood pressure (mmHg) | Visit 2 (5 min post IV) | 0.3 (6.2) | 0.5 (7.7) |
|  | Visit 2 (10 min post IV) | 1.7 (6.9) | 0.1 (6.7) |
|  | Visit 2 (20 min post IV) | 1.6 (7.4) | −1.4 (8.0) |
|  | Visit 2 (45 min post IV) | 0.3 (5.9) | 0.0 (7.7) |
|  | Visit 2 (60 min post IV) | 0.1 (6.1) | 0.7 (8.7) |
|  | Visit 2 (2 h post IV) | 0.5 (4.9) | −1.2 (8.6) |
|  | Final visit | −0.4 (5.8) | 1.6 (7.3) |
| Diastolic blood pressure (mmHg) | Visit 2 (5 min post IV) | 0.6 (5.9) | −0.2 (8.0) |
|  | Visit 2 (10 min post IV) | 1.6 (6.9) | −0.9 (7.6) |
|  | Visit 2 (20 min post IV) | 1.5 (6.4) | −0.7 (8.2) |
|  | Visit 2 (45 min post IV) | −0.2 (6.4) | 1.1 (8.1) |
|  | Visit 2 (60 min post IV) | 1.0 (5.4) | 0.3 (9.1) |
|  | Visit 2 (2 h post IV) | 1.1 (8.0) | 0.5 (9.2) |
|  | Final visit | 0.8 (9.2) | −0.1 (9.6) |
| Pulse rate (bpm) | Visit 2 (5 min post IV) | 0.7 (9.1) | −1.4 (9.5) |
|  | Visit 2 (10 min post IV) | 1.4 (8.3) | −1.5 (10.1) |
|  | Visit 2 (20 min post IV) | −0.6 (9.9) | −0.1 (9.8) |
|  | Visit 2 (45 min post IV) | −1.0 (8.1) | 0.3 (9.2) |
|  | Visit 2 (60 min post IV) | −1.3 (9.1) | 0.4 (8.8) |
|  | Visit 2 (2 h post IV) | −1.6 (10.9) | 1.0 (9.3) |
|  | Final visit | −2.2 (12.6) | 2.7 (12.0) |

Abbreviations: bpm, beats per minute; h, hours; SD, standard deviation; SS-IV, intravenous Safety Set; min, minutes.

^a^Blood pressure: n = 47 for 5 min, 10 min, 20 min, 45 min, 2 h post dose, and final visit.

^b^Pulse rate: n = 47 for final visit.

^c^Blood pressure: n = 54 for 5 min, 10 min, 20 min, 45 min, and 60 min post dose; n = 53 for 2 h post dose; n = 48 for final visit.

^d^Pulse rate: n = 54 for 5 min, 10 min, 20 min, 45 min, 60 min, and 2 h post dose; n = 48 for final visit.

## Twelve-lead ECGs

**Table S2** Mean change from baseline in 12-lead ECG parameters (SS-IV)

| **Change from baseline, mean (SD)** | | **Patients ≥1 month to  <8 years of age (N = 48)^a^** | **Patients ≥8 to  <17 years of age  (N = 55)^b^** |
| --- | --- | --- | --- |
| Heart rate (bpm) | Visit 2 (15 min post IV) | −4.0 (14.0) | 2.4 (11.7) |
|  | Visit 2 (30 min post IV) | −3.3 (14.3) | 4.9 (12.0) |
|  | Visit 2 (60 min post IV) | −5.2 (15.9) | 4.0 (13.8) |
|  | Visit 2 (2 h post IV) | −5.3 (14.9) | 4.3 (13.2) |
|  | Final visit | −4.8 (17.1) | 6.0 (12.5) |
| PR interval (ms) | Visit 2 (15 min post IV) | 3.6 (10.6) | 4.8 (11.4) |
|  | Visit 2 (30 min post IV) | 3.4 (10.6) | 3.9 (9.5) |
|  | Visit 2 (60 min post IV) | 3.5 (8.7) | 4.0 (10.7) |
|  | Visit 2 (2 h post IV) | 1.9 (12.0) | 2.4 (11.3) |
|  | Final visit | 3.2 (9.2) | 1.8 (10.4) |
| QRS interval (ms) | Visit 2 (15 min post IV) | 1.0 (4.9) | 0.4 (4.9) |
|  | Visit 2 (30 min post IV) | 0.5 (4.9) | 0.2 (5.6) |
|  | Visit 2 (60 min post IV) | 0.5 (5.1) | −0.2 (6.5) |
|  | Visit 2 (2 h post IV) | 0.1 (4.9) | 0.0 (6.1) |
|  | Final visit | 0.4 (5.0) | 0.2 (5.2) |
| QT interval (ms) | Visit 2 (15 min post IV) | 5.3 (21.7) | −0.9 (17.7) |
|  | Visit 2 (30 min post IV) | 5.1 (20.3) | −3.8 (16.4) |
|  | Visit 2 (60 min post IV) | 7.0 (23.6) | −6.6 (21.3) |
|  | Visit 2 (2 h post IV) | 5.0 (21.9) | −4.0 (21.5) |
|  | Final visit | 7.5 (22.6) | −7.6 (20.6) |
| QTcB (ms) | Visit 2 (15 min post IV) | 0.8 (21.6) | 2.9 (19.4) |
|  | Visit 2 (30 min post IV) | 0.6 (20.4) | 6.6 (19.4) |
|  | Visit 2 (60 min post IV) | 0.1 (19.8) | −0.0 (19.8) |
|  | Visit 2 (2 h post IV) | −1.7 (19.5) | 6.0 (18.5) |
|  | Final visit | 2.3 (22.0) | 5.6 (19.3) |
| QTcF (ms) | Visit 2 (15 min post IV) | 2.6 (19.7) | 1.5 (13.2) |
|  | Visit 2 (30 min post IV) | 2.3 (18.1) | 2.8 (13.2) |
|  | Visit 2 (60 min post IV) | 2.8 (18.1) | −2.5 (14.4) |
|  | Visit 2 (2 h post IV) | 1.0 (17.9) | 2.4 (13.0) |
|  | Final visit | 4.4 (18.7) | 0.9 (14.9) |
| RR duration (ms) | Visit 2 (15 min post IV) | 17.5 (64.4) | -12.2 (111.1) |
|  | Visit 2 (30 min post IV) | 19.4 (68.7) | -39.2 (104.9) |
|  | Visit 2 (60 min post IV) | 27.2 (84.7) | -24.6 (118.8) |
|  | Visit 2 (2 h post IV) | 24.5 (75.5) | -38.3 (125.2) |
|  | Final visit | 22.4 (82.4) | -53.5 (112.9) |

Abbreviations: bpm, beats per minute; ECG, electrocardiogram; h, hours; SD, standard deviation; SS-IV, intravenous Safety Set; min, minutes; ms, milliseconds; QTcB, QT interval corrected for heart rate (Bazett’s formula); QTcF, QT interval corrected for heart rate (Fridericia’s formula).

^a^n = 47 for final visit.

^b^n = 52 for 15 min post dose; n = 53 for 30 min and 60 min post dose; n = 54 for 2 h post dose; n = 47 for final visit.

# Appendix S2

## INVESTIGATOR APPENDIX

The authors acknowledge the EP0060 trial investigators for their contributions to data acquisition: Susan Arnold, MD (Children’s Medical Center Dallas, Dallas, TX, USA); Gewalin Aungaroon, MD (Cincinnati Children’s Hospital Medical Center, Cincinnati, OH, USA); Domenica Immacolata Battaglia, MD (Fondazione Policlinico Universitario Agostino Gemelli, Rome, Italy); Monika Bessenyei, MD (Debreceni Egyetem Központ, Debrecen, Hungary); Francesca Darra, MD (Azienda Ospedaliera Universitaria Integrata Verona, Verona, Italy); Dmytro Delva, MD (Ivano-Frankivsk Regional Children’s Clinical Hospital, Ivano-Frankivsk, Ukraine); Gabriella Di Rosa, MD (Azienda Ospedaliera Universitaria Policlinico G. Martino, Messina, Italy); Nicole Williams Doonan, MD (Gillette Children’s Specialty Healthcare, Saint Paul, MN, USA); Viktor Farkas, MD (Semmelweis Egyetem I.sz. Gyermekgyógyászati Klinika, Budapest, Hungary); Bernardo Flasterstein, MD (Axcess Medical Research, Loxahatchee, FL, USA); Pongkiat Kankirawatana, MD (Children’s Hospital of Alabama, Birmingham, AL, USA); Volodymyr Kharytonov, MD (Territorial Medical Association Psychiatry, Kyiv, Ukraine); Michael Kohrman, MD (Akron Children’s Hospital, Akron, OH, USA); Alla Kyrychenko, MD (Municipal Institution “Dnipropetrovsk Regional Clinical Children’s Hospital” of Dnipropetrovsk Regional Council, Dnipro, Ukraine); Volodymyr Kyrychenko, MD (Municipal Institution “Vinnytsya Regional Psychoneurological Hospital named after academician O. I. Iuschenko”, Vinnytsya, Ukraine); Liudmyla Kyrylova, MD (State Institution “Institute of Pediatrics, Obstetrics and Gynecology named after academician O. M. Lukyanova of NAMS of Ukraine”, Kyiv, Ukraine); Carlos Lastra, MD (Saint Peter’s University Hospital, New Brunswick, NJ, USA); Iryna Makedonska, MD (Municipal Institution “Dnipro City Pediatric Clinical Hospital No. 5” of the Dnipro City Council, Dnipro, Ukraine); Volodymyr Martyniuk, MD (State Institution “Ukrainian Medical Rehabilitation Centre for Children with Organic Disorders of Nervous System of the Ministry of Health of Ukraine”, Kyiv, Ukraine); Barbara Prawdzic-Seńkowska, MD (Wojewódzki Specjalistyczny Szpital Dziecięcy im. św. Ludwika w Krakowie, Krakow, Poland); Roshan Raja, MD (Child Neurology Specialists, Henderson, NV, USA); Gregory Sharp, MD (Arkansas Children’s Hospital, Little Rock, AR, USA); Jerry Tomasovic, MD (Road Runner Research, San Antonio, TX, USA).
